# Clinical impact for advanced non-small-cell lung cancer patients tested using comprehensive genomic profiling at a large USA health care system

**DOI:** 10.1016/j.esmorw.2024.100057

**Published:** 2024-07-25

**Authors:** R. Meng, A.K. Dowdell, A. Vita, D. Hanes, B. Bapat, S.-C. Chang, L. Harold, M. Schmidt, C. Wong, H. Poon, B. Schroeder, R. Weerasinghe, R. Sanborn, R. Leidner, W.J. Urba, C. Bifulco, B. Piening

**Affiliations:** 1 Providence Health, Portland; 2 Earle A. Chiles Research Institute, Portland; 3 Illumina, Inc., San Diego; 4 Microsoft Research, Redmond, USA

**Keywords:** genomics, comprehensive genomic profiling, non-small-cell lung cancer, precision therapy, patient outcomes, biomarker

## Abstract

**Background:**

New breakthroughs in precision therapies are transforming cancer care for patients with advanced non-small-cell lung cancer (NSCLC). To this effect, comprehensive genomic profiling (CGP) has emerged as a streamlined workflow to test for all relevant tumor biomarkers within a single assay. Despite this, there are still significant gaps in access to CGP testing, with many patients only tested for a subset of biomarkers or not tested at all.

**Materials and methods:**

We assessed the clinical impact of wide deployment of an in-house CGP assay at a large health care system in the United States by analyzing a cohort of advanced-stage NSCLC patients who received CGP testing and a retrospective cohort that was tested with a prior-generation 50-gene assay (small panel).

**Results:**

Seventy-seven percent of CGP-tested NSCLC patients had one or more tumor biomarkers that were actionable for a precision therapy compared to 63% of small panel-tested patients. CGP-tested patients with an actionable biomarker received appropriate precision therapies at a higher rate than small panel-tested patients (64% versus 50%, *P* < 0.001), and there were marked improvements in survival outcomes for patients tested with CGP [median overall survival (OS) 15.7 months versus 7 months, *P* < 0.0001].

**Conclusions:**

These data demonstrate clear precision therapy selection and patient OS benefits from universal access to CGP testing. Despite this, not all patients with an actionable biomarker received a precision therapy, suggesting that there are still gaps between access to CGP testing and access to precision therapies.

## Highlights

• CGP identifies more actionable alterations when compared to a prior 50-gene assay.
• CGP-tested patients received precision therapy at higher frequency than prior assay.
• When receiving CGP upon diagnosis, patients exhibited better OS.
• Despite the benefits, not all patients receive CGP testing, suggesting that gaps remain.

## Introduction

While non-small-cell lung cancer (NSCLC) remains one of the leading causes of cancer-related deaths worldwide, a remarkable therapeutic transformation is under way, with a wide variety of new precision therapeutics emerging, targeting both rare and highly common driver alterations and resulting in significant survival benefits.^1^, ^2^, ^3^, ^4^, ^5^, ^6^, ^7^ Concurrently, immunotherapeutics (IO), particularly immune checkpoint blockade therapies, have led to durable responses in a subset of patients.^8^, ^9^, ^10^, ^11^ The number of actionable molecular biomarkers in NSCLC is also rapidly expanding, including genome-wide biomarkers such as the assessment of tumor mutational burden (TMB) associated with anti-programmed cell death protein 1 IO response.^12^, ^13^, ^14^, ^15^, ^16^, ^17^, ^18^, ^19^ As such, prior testing modalities involving single gene tests and small gene panels (≤50 genes) are largely being eclipsed by the emergence of comprehensive genomic profiling (CGP) panels, which are designed to capture all actionable biomarkers in a single assay.^20^ While the majority of CGP testing has previously been carried out by large commercial laboratories or a few advanced academic cancer centers, recent drops in per-base sequencing costs and streamlined bioinformatics and reporting pipelines have enabled the adoption of in-house CGP by smaller hospital-based laboratories and academic institutions.

In this study, we assess the impact on thoracic oncology clinical practice of the adoption of in-house CGP testing in the Providence health system over a multi-year period. We evaluate the rate of actionable mutations identified via CGP testing in comparison to prior testing practices that used a 50-gene panel. We also evaluate therapy utilization in these contexts as well as survival outcome for treated patients who received CGP versus small panel testing. The goal of the study is to provide real-world results of the implementation of a CGP testing program for NSCLC in a community-based health care setting that may be of utility to hospitals/laboratories considering a similar approach in their own practices.

## Materials and methods

### Study cohorts

We evaluated test results, clinical practice decisions, and outcomes for patients diagnosed with advanced NSCLC at Providence following the introduction of in-house CGP testing in 2019. CGP testing was carried out over this time using the ProvSeq 523 assay, a 523-gene laboratory-developed procedure validated based on College of American Pathologists (CAP) standards. The assay was developed using TruSight Oncology 500 High Throughput research reagents (Illumina, San Diego, CA) and sequenced on a NovaSeq 6000 sequencer (Illumina, San Diego, CA). TMB was calculated as mutations per megabase (mut/Mb) of DNA across the sequenced space, and TMB-high was defined as ≥10 mut/Mb. The majority of patients also received programmed death-ligand 1 (PD-L1) immunohistochemistry (IHC) testing along with next-generation sequencing (NGS). Results were compared to a prior 2-year period of testing advanced NSCLC patients using a prior 50-gene test.

### Institutional review board approval

All research was carried out under institutional review board (IRB) protocol 201900048 “Effect of automatic reflex genomics testing on clinical and economic outcomes in cancer” approved by the Providence IRB. All CGP results and associated clinical metadata were deidentified and aggregated for these analyses.

### Clinico-genomic dataset construction

We created an integrated dataset with curated and standardized fields. Biomarker data included variant call files, annotations, designations of pathogenicity by pathologists/geneticists, clinical interpretations, sequencing quality metrics, and PD-L1 IHC results. Biomarker results were linked to clinical data extracted from both structured and unstructured electronic health records [Epic Care Everywhere (EHRs)]. Some data points (e.g. histology, treatment status) were curated manually.

### Data extraction and utilization of Snowflake

Electronic medical records from across clinical sites submitting samples and genomic data were curated, standardized, and stored on the cloud data warehouse. Patients who received CGP testing between September 2019 and November 2021, and small panel testing between January 2017 and November 2018, were selected for further analysis. Between November 2018 and September 2019, patients received testing utilizing the TruSight Tumor 170 assay (Illumina), but due to the low number of tests provided and the protocol-driven nature of the test, no tests from this time frame were analyzed.

### Natural language processing-based chart mining

To extract PD-L1 IHC results from clinical notes, we developed a natural language processing (NLP)-based information extraction system. Our system uses a customized version of spaCy^21^ for sentence segmentation and NLTK^22^ for tokenization. For key result extraction, our system uses domain-specific rules to identify relevant entities and potential cross-sentence relations, as well as to determine whether they are positive assertions. Each extracted result contains the PD-L1 gene mentions, assay types (22C3, 28-8, SP263, SP142), staining intensity, analysis methods (tumor proportional score or combined positive score), and expression values (e.g. <1%, 10, high). We used the note date as a proxy for the measurement date, but we plan to extract the measurement date from the note text in future work. Our pipeline runs daily on new pathology reports, imaging reports, progress notes, encounter notes, operative notes, and surgery notes, but we find that most PD-L1 results are reported in pathology reports and progress notes.

We developed our rules using notes before a certain date and constructed a test set from pathology reports and progress notes after that date. For the test set, we randomly selected 135 cancer patients and sampled one note per patient that contains a PD-L1 mention in any of its surface forms (e.g. PDL1, PDL-1, PDL 1). We asked an expert to manually annotate each sampled note for PD-L1 expression values and the analysis methods. We evaluated our pipeline by comparing the extracted expression values and methods with the expert annotations. The evaluation results show that our pipeline achieved high performance: precision 97.5%, recall 89.6%, and F1 93.4%.

### Actionability assessment

Actionability was assessed based on criteria compiled in the OncoKB database,^23^ a curated database that classifies all mutations into tumor-specific tiers of actionability. Briefly, tumor types were translated to OncoTree codes, assessed for stage, and matched to actionable biomarker categories (OncoKB levels 1 and 2). In order to characterize the subset of cases with potentially actionable markers for study, we compiled all reported pathogenic variants (e.g. single nucleotide variants, indels, fusions, copy number variants) from the cohort and assessed potential actionability based on OncoKB therapeutic levels of evidence.^23^ Patients were assigned to the level of most significant alteration based on levels of evidence: actionable biomarkers predictive of response to Food and Drug Administration (FDA)-approved therapies (level 1), or predict response to guideline-recommended, standard-of-care therapies (level 2). In instances where a biomarker became actionable within the window of our study (Supplementary Table S1, available at https://doi.org/10.1016/j.esmorw.2024.100057), *ALK* oncogenic mutations in November 2018, *RET* fusions in November 2018, *NTRK* fusions in November 2018, *EGFR* exon 20 insertions in May 2021, and *KRAS G12C* in May 2021, patients who received genetic testing before the FDA approval date of the specific treatment were not considered actionable, even if they harbored the specific alteration. To compare the CGP- and small panel-tested cohorts more accurately, we carried out an additional analysis of the CGP cohort limiting the analysis to only capture alterations actionable before 2018.

### Treatment selection

We assessed therapy selection after CGP testing among patients with metastatic (stage IV) NSCLC who were followed up at Providence for their care. Treatments were classified by type of therapy: targeted therapy (TT), IO, chemotherapy and IO (chemo + IO), chemotherapy only, and targeted therapy off-label (TTOL). Individual patients were assessed for the actionable biomarkers that they possessed, along with the treatment that they received and visualized in R v4.2.2 using the UpSetR package.

### Clinical outcomes

Downstream clinical outcomes—including overall survival (OS)—were documented among patients with available follow-up at Providence. OS was calculated as the duration in months from the date of report for most recent genetic testing to the date of last follow-up or death from any cause. OS by treatment type is presented in unadjusted Kaplan–Meier figures and subsequently tested using a Cox proportional hazards model adjusted for patient age and tumor type. All survival analyses were carried out in R v4.2.2 using the survival package.

## Results

In this study, 759 patients comprised the CGP cohort versus 192 patients in the small panel cohort (Supplementary Figure S1, available at https://doi.org/10.1016/j.esmorw.2024.100057). All patients were tested with in-house laboratory-developed panels (CGP or small panel). Patients in both cohorts had a median age of 69 years, were 82% white, and majority were non-Hispanic non-Latino (Table 1). A higher proportion of males were tested in the CGP cohort compared to the small panel cohort (51% versus 42%, *P* < 0.04). Notably, as described in the Materials and methods section, the years of testing for the small panel cohort were 2017-2018 and the years of testing for the CGP cohort were 2019-2021.

**Table 1:** Patient characteristics

*n*	CGP	Small panel	*P*
	**759**	**192**	
Age at time of testing, median (IQR), years	69.0 (63.0-77.0)	69.0 (62.0-77.0)	0.569
Sex, *n* (%)			0.033
Female	375 (49.4)	112 (58.3)	
Male	384 (50.6)	80 (47.4)	
Race, *n* (%)			0.592
White or Caucasian	621 (81.8)	157 (81.8)	
Asian	40 (5.3)	13 (6.8)	
Black or African American	17 (2.2)	5 (2.6)	
American Indian or Alaska Native	13 (1.7)	1 (0.5)	
Native Hawaiian or Other Pacific Islander	6 (0.8)	0 (0.0)	
Unknown	62 (8.2)	16 (8.3)	
Ethnicity, *n* (%)			0.477
Hispanic or Latino	24 (3.2)	4 (2.1)	
Not Hispanic or Latino	696 (91.7)	180 (93.8)	
Not specified	24 (3.2)	7 (3.6)	
Unknown	15 (2.0)	1 (0.5)	
Stage, *n* (%)			<0.001
Stage III	204 (26.9)	12 (6.2)	
Stage IV	555 (73.1)	180 (93.8)	
Year of test ordered, *n* (%)			
2017	0 (0.0)	111 (57.8)	
2018	0 (0.0)	81 (42.2)	
2019	29 (3.8)	0 (0.0)	
2020	338 (44.5)	0 (0.0)	
2021	392 (51.6)	0 (0.0)	
Turnaround time in days^a^ median (IQR)	14.0 (11.0-15.0)	11.0 (8.0-14.0)	<0.001
Vital status, *n* (%)			<0.001
Alive	385 (50.7)	33 (17.2)	
Deceased	374 (49.3)	159 (82.8)	
Follow-up in months since testing ordered,^b^ median (IQR)	11.0 (3.0-19.0)	7.0 (2.0-24.3)	0.876

Characteristics of tested advanced-stage NSCLC patients are presented.

CGP, comprehensive genomic profiling; IQR, interquartile range.

Source: Providence Health System, 2017-2021.

a *n* = 3 missing values were excluded.

b *n* = 2 missing values were excluded.

**Figure 1:**
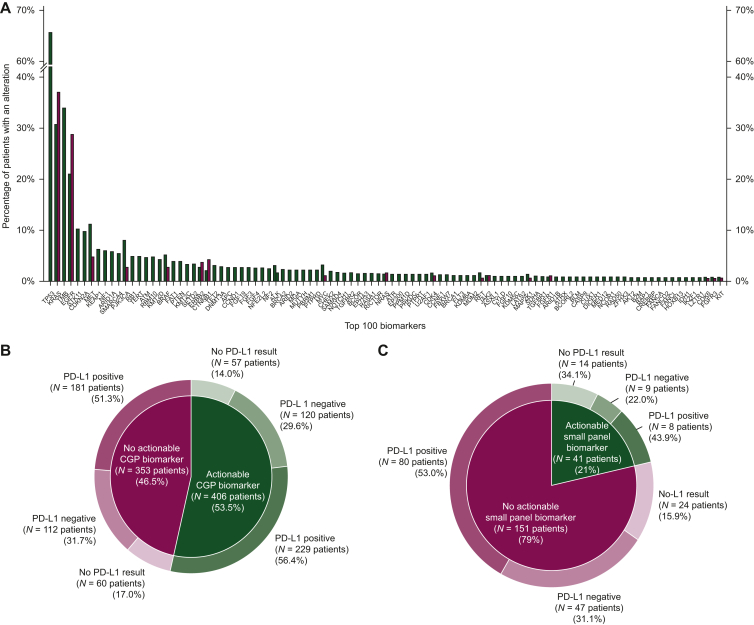
**Biomarker spectrum across the cohort.** (A) Frequency of pathogenic alterations detected via CGP (green) versus small panel (burgundy). CGP biomarker actionability and PD-L1 IHC results (B) for the CGP-tested cohort (*n* = 759 patients) and (C) for the small panel-tested cohort (*n* = 192 patients). CGP, comprehensive genomic profiling; IHC, immunohistochemistry; PD-L1, programmed death-ligand 1.

We next assessed pathogenic and actionable marker detection across both cohorts. Figure 1 shows the frequency of detection of pathogenic alterations across each cohort. One or more pathogenic alterations were identified in 587 (77%) CGP-tested patients (Figure 1B) compared to 121 (63%) small panel-tested patients (Figure 1C) (*P* < 0.0001). Of those patients with a pathogenic alteration, 406 (53%) CGP-tested patients and 41 (21%) small panel-tested patients were harboring an actionable alteration based on OncoKB assessment (*P* < 0.00001). Due to the testing period discrepancy between the two panels, we also analyzed the CGP cohort only looking at alterations that would have been actionable between 2017 and 2018. If the CGP testing had been carried out during that time frame, actionable alterations would have been identified in 167 patients (22%), reflecting both the increase in actionable biomarkers over time and the ability of CGP to detect biomarkers not assessed via small panels (such as TMB). Key differences in biomarkers detected were high rates of *TP53*, *STK11*, and *CDKN2A* mutations and high TMB, which were detected in CGP but were not part of the genes/biomarkers comprising the small panel. Patients in both cohorts had similar rates of PD-L1 positivity, with 63.8% of CGP-tested patients and 63.6% of small panel-tested patients being PD-L1 positive. The most frequent actionable alterations in CGP were TMB-high, followed by *EGFR* and *MET*. The most frequent alterations for small panel testing were *EGFR*, *MET*, and *ALK*, as TMB-high was the only National Comprehensive Cancer Network (NCCN) guideline biomarker not tested by the small panel.

**Figure 2:**
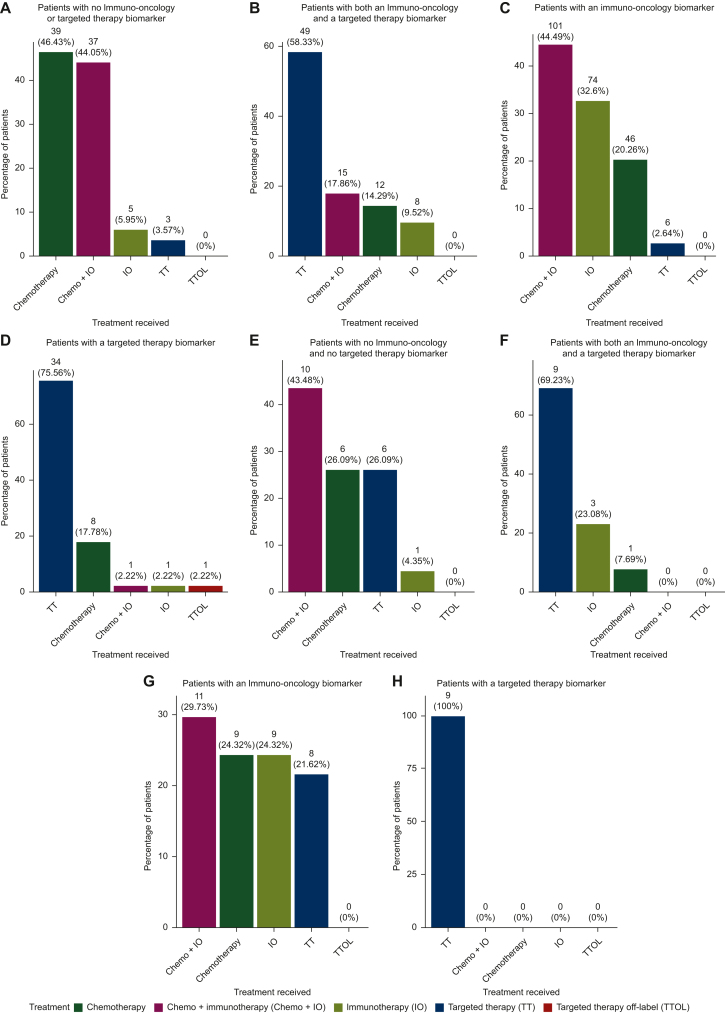
**Therapy utilization for patients with different biomarker profiles.** (A-D) Chemotherapy, IO, chemo + IO, TT, and TTOL use for patients with or without actionable TT or IO biomarkers for CGP-tested patients. (E-H) The same assessment for patients tested with the small panel is shown. CGP, comprehensive genomic profiling; chemo, chemotherapy; IO, immunotherapy; TT, targeted therapy; TTOL, targeted therapy off-label.

While CGP testing revealed more actionable alterations, this may not necessarily translate to increased utilization of precision therapies. As such, we next carried out EHR chart mining (both NLP based and manual) to assess rates of therapy use for the CGP and small panel cohorts. From the initial cohorts of tested patients, we identified 440 CGP-tested patients and 82 small panel-tested patients with sufficient follow-up records, who were included in the below assessments. Overall, 64% (*n* = 281 patients) of CGP-tested patients with an actionable biomarker received an appropriate precision therapy. Patients who harbored both an actionable biomarker for a TT and IO received an appropriate precision treatment 86% of the time (*n* = 72 patients) (Figure 2B). Of note, the therapy selection in patients with both a TT and an IO biomarker was more frequently TT than IO (49 patients versus 23 patients, respectively). Patients who possessed either an actionable TT or IO biomarker received appropriate precision therapies at similarly high rates, with 77% of patients with an IO biomarker and 76% of patients with a TT biomarker receiving the correct precision therapy (Figure 2C and D). Small panel-tested patients who had both IO and TT biomarkers received an appropriate precision therapy 92% of the time (*n* = 13 patients) (Figure 2F). When comparing treatment received by patients who had an eligible TT or IO biomarker, all nine patients who harbored only a TT biomarker received an appropriate therapy. There was, however, a stark difference in patients with an IO biomarker with only 54% of small panel-tested patients receiving the correct treatment (Figure 2G and E); this is likely reflective of the less widespread use of IO clinically in the 2017-2018 time frame. Most patients with no actionable biomarkers received either chemotherapy alone or a combination of chemo + IO (Figure 2A and E). Overall, use of TT in patients who did not possess an actionable biomarker was very low with only three and six patients in the CGP and small panel cohorts, respectively, receiving a TT.

**Figure 3:**
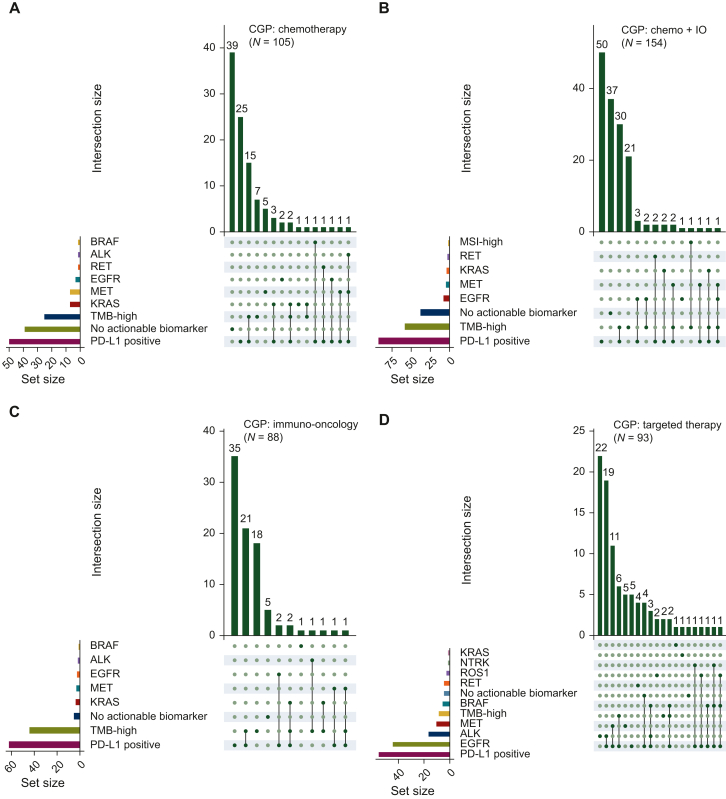

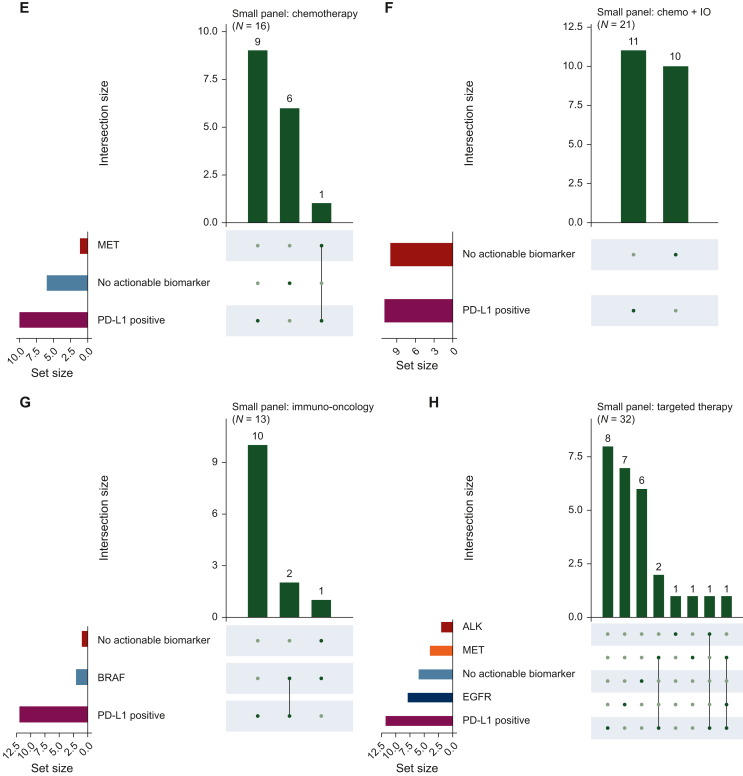
**UpSet plot showing treatment utilization for patients with specific TT/IO biomarkers.** (A-D) Biomarker associations for patients treated with chemotherapy, chemo + IO, IO, and TT in the CGP cohort are shown. (E-H) The same breakdown for patients in the small panel cohort is shown. CGP, comprehensive genomic profiling; chemo, chemotherapy; IO, immunotherapy; MSI, microsatellite instability; PD-L1, programmed death-ligand 1; TMB, tumor mutational burden; TT, targeted therapy.

We next carried out a deeper dive into these interesting treatment patterns, specifically looking at which specific markers were or were not acted upon clinically. As shown in Figure 3, patients with single biomarker results for highly actionable biomarkers largely received the appropriate TT (e.g. *EGFR*, *ALK*, *ROS1*, *NTRK1*) (Figure 3D and H). Similarly, for the patients who received IO or a combination of chemo + IO, the majority of patients harbored an actionable IO biomarker [e.g. TMB-high, microsatellite instability (MSI)-high, PD-L1 positive] (Figure 3B and F, 3C and G). There were outliers to this, with multiple patients with an *EGFR* mutation receiving chemotherapy and/or IO. Outliers were also observed for patients with *MET*, *ALK*, and *RET.*

**Figure 4:**
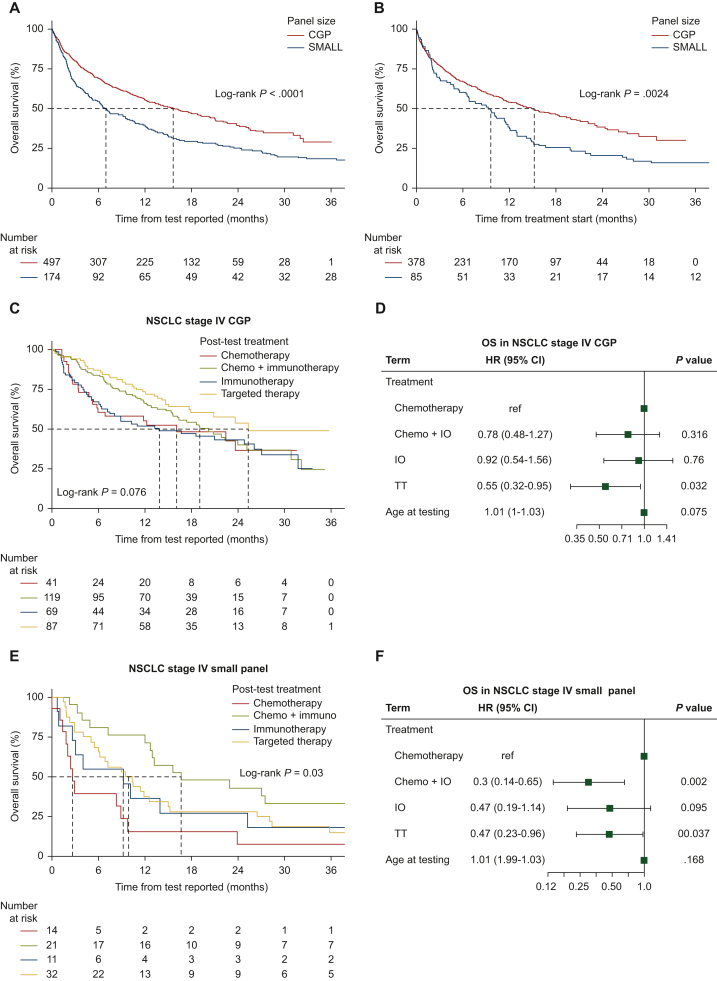
**Survival analysis of CGP- and small panel-tested patients.** (A) Kaplan–Meier survival analysis of the CGP versus small panel cohorts based on time from initial test report date. (B) Kaplan–Meier survival analysis of the CGP versus small panel cohorts based on time from treatment start. (C) Survival analysis for CGP-tested patients by treatment type. (D) Hazard analysis for CGP-tested patients. (E) Survival analysis for small panel-tested patients by treatment type. (F) Hazard analysis for small panel-tested patients. CGP, comprehensive genomic profiling; chemo, chemotherapy; CI, confidence interval; HR, hazard ratio; NSCLC, non-small-cell lung cancer; OS, overall survival.

Having observed that CGP utilization is associated with increased detection of actionable biomarkers and precision therapy utilization, we next asked whether this translated into improved outcomes for the patient population. As such, we carried out survival analysis on the CGP and small panel cohorts both overall and when stratified by biomarker presence (Figure 4). Comparing OS for the CGP versus small panel cohorts, we see a significant survival advantage in the CGP cohort (Figure 4A, *P* < 0.0001), with a median OS in the CGP cohort of 15.7 months [95% confidence interval (CI) 12.4-20.2 months] versus 7.0 months (95% CI 5.1-10.4 months) for the small panel cohort. We also assessed OS in both cohorts utilizing the time to first treatment, as opposed to the time of test report date, and found the results to again show a significant survival advantage in the CGP cohort (Figure 4B, *P* = 0.0024). Looking at survival by treatment type, we see that TT use in CGP-tested patients (*n* = 87 patients; 28% of patients with sufficient follow-up) was associated with significantly higher survival as compared to chemotherapy [Figure 4C and D, median OS 25.5 months [95% CI 21.0 months-infinity (Inf)] versus 16.2 months (95% CI 5.91 months-Inf), *P* = 0.03], also see Supplementary Figure S2, available at https://doi.org/10.1016/j.esmorw.2024.100057. We observed a similar survival advantage for TT versus chemotherapy in the small panel cohort [Figure 4E and F, median OS 10.18 months (95% CI 6.3-15.6 months) versus 2.8 months (95% CI 2.1 months-Inf), *P* = 0.04]; however, in the small panel cohort, only 32 patients (41%) received a TT. Interestingly, the chemo + IO combination showed a significant survival advantage in the small panel cohort versus chemotherapy alone (*P* = 0.002), whereas it was not observed to be significantly different from chemotherapy in the CGP cohort. OS between patients who received chemotherapy alone differed significantly between the CGP cohort and the small panel cohort (median OS 16.2 months versus 2.8 months).

## Discussion

While this study and others show a clear benefit for a precision-therapy-guided care path for patients with advanced NSCLC, there is still tremendous variability in utilization of genomic testing in NSCLC treatment guidance, CGP or otherwise. Recent data remarkably show that the proportion of advanced/metastatic NSCLC patients who received biomarker testing by NGS-based methods rose from 28% in 2015 to 68% in 2020.^24^ Despite the increase in test utilization, nearly one-third of patients still did not receive an NGS-based biomarker test. Currently identified barriers to testing include cost of testing, physician unfamiliarity with testing strategies, patient lack of insurance, or insurance denials for testing. As such, patients from socioeconomically disadvantaged backgrounds likely do not have the same access to precision-guided care paths. Given the significant survival benefits afforded by these care paths, a significant investment is required across the field in both identifying and closing these gaps.

While CGP has become more widely adopted in recent years, single gene and small panel testing are still more prevalent for NSCLC patient testing. A recent study highlighted that while 90% of patients in their cohort received at least one single biomarker test, only 35% received all five tests for individual biomarkers before first-line (1L) therapy.^25^ Previous studies have also shown that patients who receive biomarker testing and those who receive their testing before 1L therapy both have reduced adjusted hazard of death^26^ and that patients who receive a TT exhibit better OS compared to those who received a non-TT.^27^ These studies, along with ours, highlight the benefit of early and expansive testing, and that a significant benefit to the overall field can be achieved by enabling better access to CGP testing. One such strategy is democratizing access to CGP testing to in-hospital laboratories around the world. While there are a variety of commercial laboratories that carry out CGP testing on a send-out basis, many oncologists prefer to use their in-house laboratories due to close communication/collaboration with in-house molecular pathology teams, ease of tissue procurement for sequencing, potential for faster turnaround time, and availability of downstream NGS data for research. As such, strategies such as co-panel development, open-source bioinformatics pipelines, and decreased sequencing costs can enable a variety of clinical settings of various sizes to offer in-house CGP as an option. Ultimately, universal access to CGP testing either through in-house or commercial offerings remains a key goal to improve precision medicine adoption and ultimately improve patient outcomes.

While our study highlights the benefits of CGP testing for advanced-stage NSCLC patients, there are some limitations. One caveat is that our study may not have sufficient follow-up time in order to measure the characteristic long tail of durable survival in a subset of IO-treated patients. As such, the survival gains we see in the CGP cohort appear to be largely driven by increased utilization of TT. Additionally, the two cohorts were tested during different time periods (CGP 2019-2021, small panel 2017-2018), which limits our ability to compare the two due to the approval of new treatments and actionable biomarkers. Despite the difference in the testing time period for the two methods, CGP does have novel biomarker signatures such as TMB and MSI that are not typically tested in small panels, and those biomarkers would have not been analyzed even if the testing took place at a later date. We also observed significant differences in the OS in patients treated with chemotherapy between the two testing methods. We believe this is due to the fact that CGP testing was implemented under a pathologist-led protocol whereby testing was most often ordered at the time of advanced cancer diagnosis, where historical small panel ordering sometimes took place before different lines of therapy.

A key observation from this study was that while the majority of patients with an actionable biomarker received the associated precision therapy, we observed a variety of gaps where patients harboring a potentially actionable marker received conventional chemotherapy or IO. There may be a variety of reasons for this, including lack of access to the precision drug due to cost and/or availability, patient refusal for other reasons, or physician unfamiliarity with the biomarker/drug relationship. To address the latter, we have recently introduced a system-wide molecular tumor board as one strategy to (i) provide a panel of precision medicine experts to assist with treatment decisions, and (ii) provide physician education through real-world examples of precision therapy treatments and associated outcomes. We expect that this and other educational opportunities may increase precision medicine adoption, and a key follow-up study will be to assess the impact of these programs over time on precision therapy utilization. Moreover, a current study under way is focused on understanding which key populations across our diverse health system are most affected by gaps in access to testing and or precision therapies so that we can develop new programs that enable access to precision medicine for all.
