# Supplementary material for: Clinical impact for advanced non-small-cell lung cancer patients tested using comprehensive genomic profiling at a large USA health care system
Source: ESMO Real World Data Digit Oncol. 2024 Jul 25;5:100057. doi: 10.1016/j.esmorw.2024.100057 (PMC12836689; doi:10.1016/j.esmorw.2024.100057)
Supplement: Supplementary Table I [file mmc1.docx]

**Supplemental Table I. Table of Actionable Alterations.** List of OncoKB level 1 and 2 actionable alterations in NSCLC. Table contains the drugs used to treat those specific alterations, the FDA approval date of the drug (where applicable), the treatment category, and the level of evidence.

| **Biomarker (Alteration)** | **Drug(s)** | **FDA Approval Date** | **Treatment Category** | **ONCOKB Level of Evidence** |
| --- | --- | --- | --- | --- |
| ALK (Fusions) | Alectinib \| Brigatinib \| Ceritinib \| Crizotinib | 12/11/2015 \| 04/28/2017 \| 04/29/2014 \| 08/26/2011 | TT | Level 1 |
| ALK (Oncogenic Mutations) | Lorlatinib | 11/2/2018 | TT | Level 1 |
| BRAF (V600E) | Dabrafenib + Trametinib | 7/15/2015 | TT | Level 1 |
| EGFR (Exon 19 Deletion, L858R) | Afatinib \| Dacomitinib \| Erlotinib \| Erlotinib + Ramucirumab \| Gefitinib \| Osimertinib | 7/12/2013 \| 09/27/2018 \| 11/18/2004 \| 05/29/2020 \| 05/05/2003 \| 04/18/2018 | TT | Level 1 |
| EGFR (Exon 20 Insertion) | Amivantamab \| Mobocertinib | 05/21/2021 \| 09/15/2021 | TT | Level 1 |
| EGFR (G719, L861Q, S768I) | Afatinib | 7/12/2013 | TT | Level 1 |
| EGFR (T790M) | Osimertinib | 11/13/2015 | TT | Level 1 |
| KRAS (G12C) | Sotorasib | 5/28/2021 | TT | Level 1 |
| MET (D1010, Exon 14 Deletion, Exon 14 splice mutation) | Capmatinib \| Tepotinib | 05/06/2020 \| 02/03/2021 | TT | Level 1 |
| NTRK1/NTRK2/NTRK3 (Fusions) | Entrectinib \| Larotrectinib | 8/15/2019 \| 11/26/2018 | TT | Level 1 |
| TMB-H/MSI-H | Pembrolizumab | 5/10/2017 | IO | Level 1 |
| RET (Fusions) | Pralsetinib \| Selpercatinib | 09/04/2020 \| 05/08/2020 | TT | Level 1 |
| ROS1 (Fusions) | Crizotinib \| Entrectinib | 03/11/2016 \| 8/15/2019 | TT | Level 1 |
| EGFR (A763_Y764insFQEA) | Erlotinib | N/A | TT | Level 2 |
| ERBB2 (Oncogenic Mutations) | Ado-Trastuzumab Emtansine \| Trastuzumab Deruxtecan | N/A | TT | Level 2 |
| MET (Amplification, D1010, Exon 14 Deletion, Exon 14 Splice Mutation) | Crizotinib | N/A | TT | Level 2 |
| RET (Fusions) | Cabozantinib | N/A | TT | Level 2 |
