# Supplementary material for: Clinical impact for advanced non-small-cell lung cancer patients tested using comprehensive genomic profiling at a large USA health care system
Source: ESMO Real World Data Digit Oncol. 2024 Jul 25;5:100057. doi: 10.1016/j.esmorw.2024.100057 (PMC12836689; doi:10.1016/j.esmorw.2024.100057)
Supplement: Supplementary Figure S1 [file mmc2.pdf]

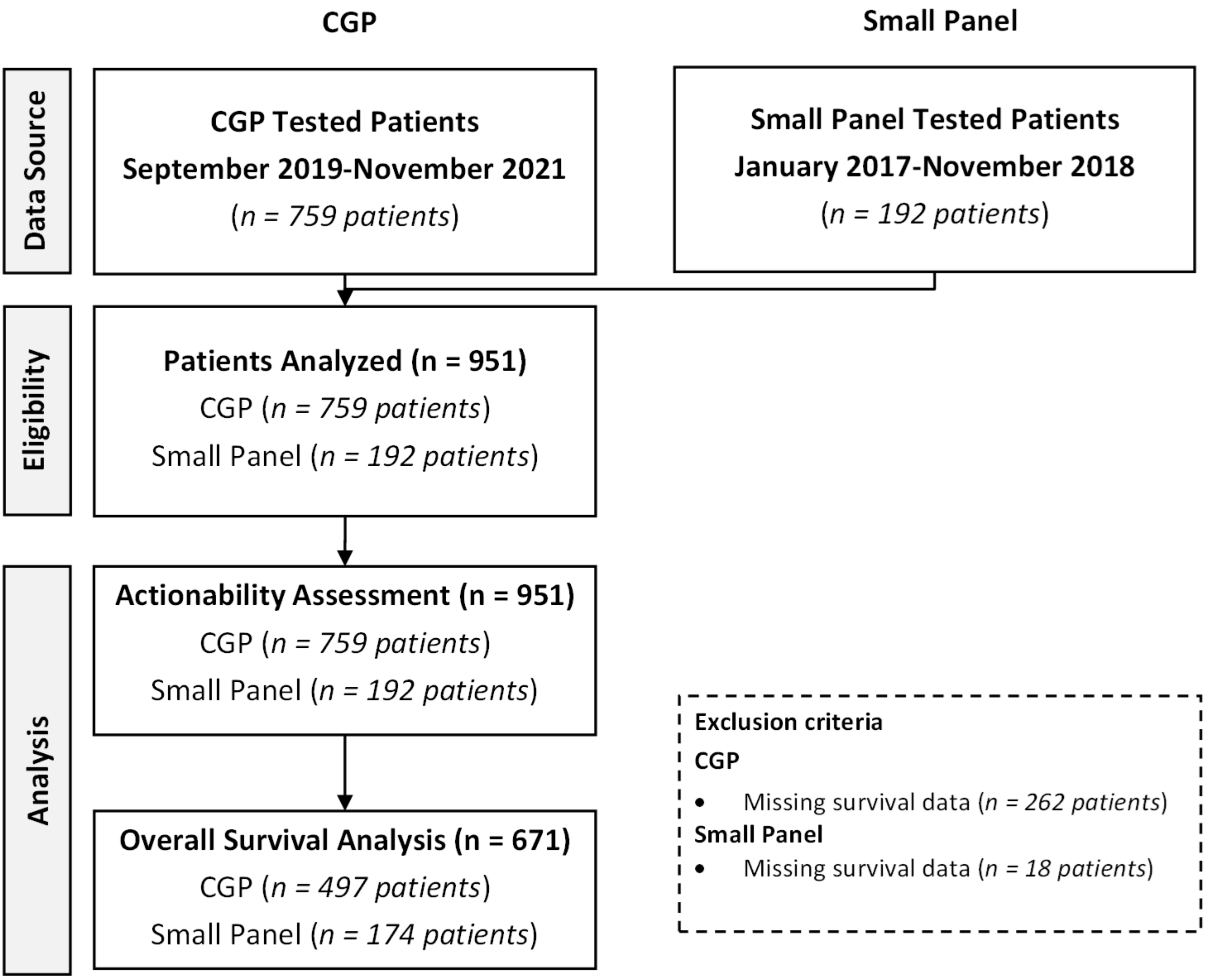

Figure S1. Flow diagram of CGP and Small Panel tested cohorts and the number of patients analyzed for actionability and overall survival.
