# Supplementary material for: Clinical impact for advanced non-small-cell lung cancer patients tested using comprehensive genomic profiling at a large USA health care system
Source: ESMO Real World Data Digit Oncol. 2024 Jul 25;5:100057. doi: 10.1016/j.esmorw.2024.100057 (PMC12836689; doi:10.1016/j.esmorw.2024.100057)
Supplement: Supplementary Figure S2 [file mmc3.pdf]

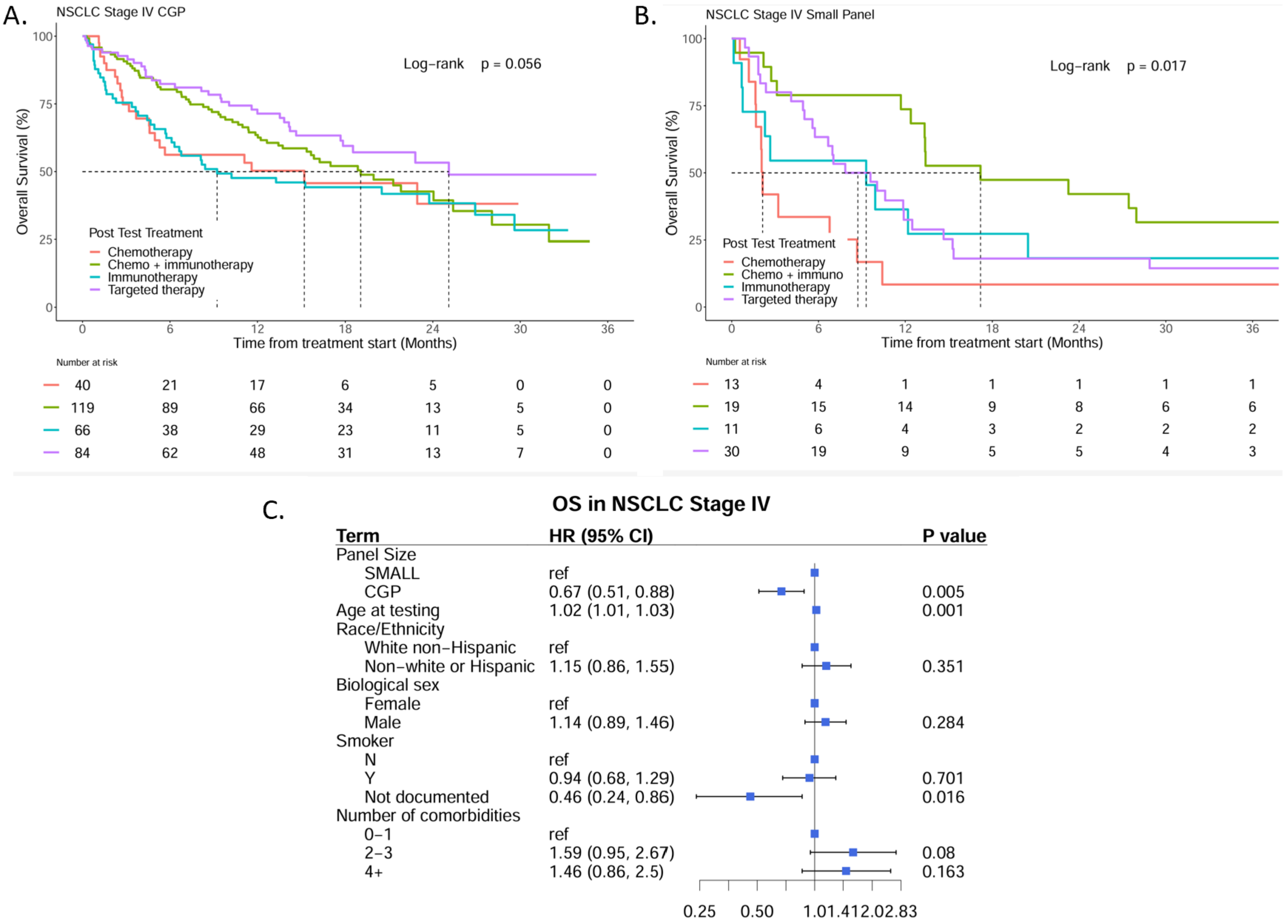

Figure S2. A) Kaplan-Meyer survival analysis of the CGP cohort, stratified by treatment type (time from treatment start). B) Kaplan-Meyer survival analysis of the Small Panel cohort, stratified by treatment type (time from treatment start). C) Hazard analysis for all NSCLC patients.
